# Intraspecific differences in plant functional traits are related to urban atmospheric particulate matter

**DOI:** 10.1186/s12870-021-03207-y

**Published:** 2021-09-22

**Authors:** Jiyou Zhu, Chengyang Xu

**Affiliations:** Research Center for Urban Forestry, The Key Laboratory for Silviculture and Conservation ofMinistry of Education, Key Laboratory for Silviculture and Forest Ecosystem of State Forestry and Grassland Administration, Beijing Forestry University, Beijing, 100083 China

**Keywords:** Leaf functional traits, Leaf economics spectrum, Atmospheric particulate matter, Trade-off

## Abstract

**Background:**

Functional trait-based ecological research has been instrumental in advancing our understanding of environmental changes. It is still, however, unclear how the functional traits of urban plants respond to atmospheric particulate matter, and which trade-off strategies are shown. In order to explore the variation of plant functional traits with the gradient of urban atmospheric particulate matter, we divided atmospheric particulate matter into three levels according to road distance, and measured the variation of six essential leaf functional traits and their trade-off strategies.

**Results:**

Here, we show that the functional traits of plants can be used as predictors of plant response to urban atmospheric particulate matter. Within the study, leaf thickness, leaf dry matter content, leaf tissue density, stomatal density were positively correlated with atmospheric particulate matter. On the contrary, chlorophyll content index and specific leaf area were negatively correlated with atmospheric particulate matter. Plants can improve the efficiency of gas exchange by optimizing the spatial distribution of leaf stomata. Under the atmospheric particulate matter environment, urban plants show a trade-off relationship of economics spectrum traits at the intraspecific level.

**Conclusion:**

Under the influence of urban atmospheric particulate matter, urban plant shows a “slow investment-return” type in the leaf economics spectrum at the intraspecific level, with lower specific leaf area, lower chlorophyll content index, ticker leaves, higher leaf dry matter content, higher leaf tissue density and higher stomatal density. This finding provides a new perspective for understanding the resource trades-off strategy of plants adapting to atmospheric particulate matter.

## Background

In a world of increasing urbanization, atmospheric particulate matter (PM) pollution mitigation is currently one of the most critical city planning issues. Urban trees are of central importance for this issue because they facilitate the deposition of various gases and particles and affect microclimate and air turbulence [1–4]. The urban PM contains harmful components such as heavy metals, which seriously affect the health of urban residents [4, 5]. In China, the government attaches great importance to the prevention and control of air pollution. In recent years, despite the continuous improvement of the ambient air quality in China, the treatment effect is unstable, especially in the autumn and winter in the Beijing-Tianjin-Hebei region and its surrounding areas. Data show that in the autumn and winter of 2018–2019, the average concentration of PM2.5 in Beijing-Tianjin-Hebei and its surrounding areas increased by 6.5% year-on-year, and the number of days of heavy pollution increased by 36.8% year-on-year [6, 7]. Due to the extraordinary terrain and meteorological conditions, Beijing has become the concentration center of air pollutants in North China [8, 9]. In addition, the acceleration of urbanization and the rapid development of traffic also bring about the prevention and control of air pollution.

At present, it is impossible to rely on pollution sources control to solve environmental problems entirely. The natural removal mechanism is an effective way to alleviate the pressure of urban air pollution, and urban trees are the critical factor [10–12]. Urban plants, as an important part of the urban ecosystem, can not only beautify the environment, but also effectively retain and adsorb particulate matter in the atmosphere [13–17]. Scholars have carried out extensive research, precisely because the urban forest has produced huge dust reduction benefits [14, 16]. Previous studies have pointed out that in 1995, the total amount of dust trapped in the green space of residential areas in Beijing suburbs was 2170 t, and in 2017, the amount of PM2.5 trapped in the green spaces of Shanghai was about 3533 t. Urban vegetation in London area can reduce the PM10 content in the air by 0.7 to 2.6% [18–21]. At present, the research on plant dust deposition mainly focuses on the comparison of deposition capacity, deposition mechanism, dust composition and deposition characteristics, and has achieved many research results [10, 22–24]. Is it worth noting that the retention of atmospheric particles in plant leaves will affect its growth and development? Do different dust deposits have different effects on plants? There is still a big gap in the research on this problem.

In the long process of evolution and development, plants interact with the environment, and gradually form a variety of adaptation strategies in internal physiology and external morphology, minimizing the adverse effects of the environment [25–29]. In the past 20 years, new concepts and measurement methods related to plant traits have emerged constantly, and their application fields have been expanding. The scientific research involves many aspects of ecological research [30]. One of the key hot issues is the relationship between plant functional traits and environment, and how the environment affects plant functional traits, thus establishing the close relationship between environment and ecosystem functions. The leaf is an important organ for plants to obtain energy, resources and nutrition, and it is also the most sensitive organ to environmental change and has strong plasticity [31, 32]. Leaf functional traits can objectively reflect the influence of environmental changes on plants and the adaptability of plants to the environment, and predict the characteristics of plants and urban ecosystems [33–35]. At present, the research on leaf functional traits and environment mainly focuses on climate (such as temperature, precipitation, light, etc.), topography (such as geographic spatial gradient variation, altitude, slope direction, etc.), nutritional status, biological invasion and land use [25–29, 36–40]. However, there are few studies on the effects of urban atmospheric particulates matter on plant functional traits.

*Euonymus japonicus* is one of the most planted landscaping trees species in Beijing, and it plays an important role in urban ecological benefits and social benefits. A study on the interception ability of 29 kinds of urban greening plants in Beijing shows that the interception ability of *Euonymus japonicus* to PM2.5 in shrubs is the second only to *Buxus microphylla* [10]. Based on this, this study takes *Euonymus japonicus* as the research object, taking the leaf functional traits as the breakthrough point. The overall goal is to study the relationship between leaf functional traits and atmospheric particulate matter. The specific objectives are as follows, (1) Evaluate the response and trade-offs of plant leaf traits to different dust deposition under the influence of atmospheric particulate matter. (2) Whether the relationship of leaf traits accords to the global leaf economics spectrum (intraspecific level) in the environment polluted by atmospheric particulate matter. Plants can adapt to environmental changes by adjusting and changing some of their functional traits, and form different survival strategies such as growth, reproduction and defense. Studying on the relationship between plant and environment based on plant functional traits can better reveal the response and adaptation rules of plants to the environment. Therefore, studying the response of plant functional traits to atmospheric particulate matter (differences, the relationship among functional traits and the balance of traits) is helpful to understand the adaptation strategies of plants to atmospheric particulate matter. This also provides an important scientific basis for the management of urban plants.

## Results

### Leaf dust deposition under different levels of atmospheric particulate matter

**Fig. 1:**
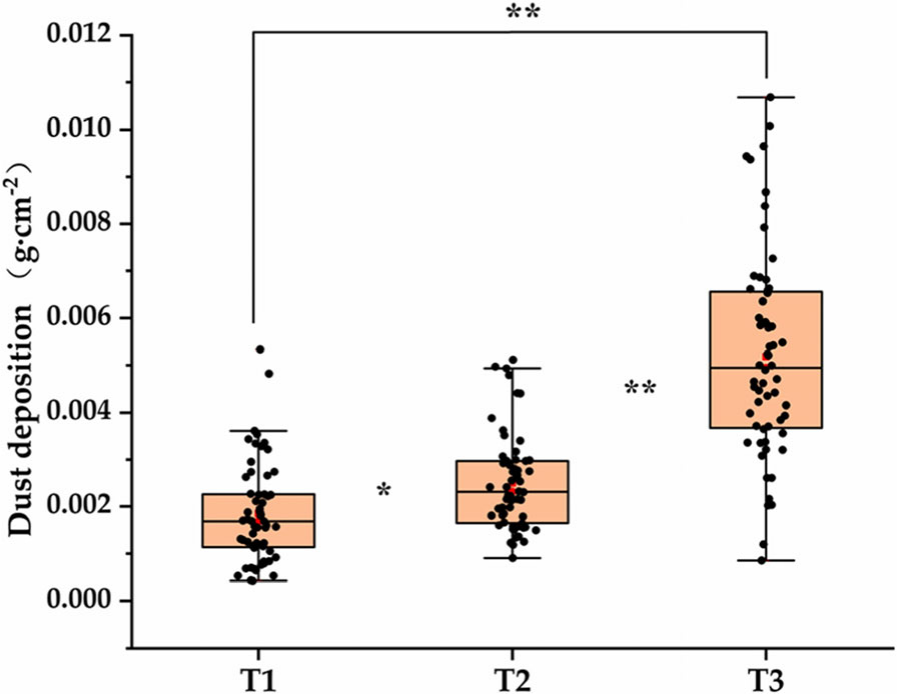
Dust deposition of plant leaves at different locations on urban streets. “*” indicate that the indicators have reached a significant difference at the *p* < 0.05 level, and “**” indicate that the indicators have reached a significant difference at the *p* < 0.01 level. T1: outer sidewalk area (slightly polluted area of atmospheric particulate matter), T2: area between the relief road and the sidewalk (moderately polluted area of atmospheric particulate matter), T3: area in the middle of the main road (heavy pollution area of atmospheric particulate matter)

As shown in Fig. 1, in three different locations of urban highway, the dust collection capacity of *Euonymus japonicus* leaves gradually decreases along the center of the road (main road) to both sides. We found that the dust deposition of *Euonymus japonicus* leaves in the middle of the main road (T3) was (0.0052 ± 0.0022 g) > that between the relief road and the sidewalk (T2) (0.0025 ± 0.001 g) > that of the outer sidewalk (T1) (0.0019 ± 0.001 g). On the main road, on the one hand, cars emit a lot of exhaust gas, on the other hand, they also cause a lot of dust. Because the dust on the ground was driven by the air flow of vehicles, it was an important part of the total suspended particulate matter in the ambient air. Such atmospheric particulate matter mainly diffuses to both sides. The auxiliary road vehicles were mainly non-motor vehicles, and the traffic volume was small, and the suspended particulate matter in the atmosphere was relatively low. However, the main body of the sidewalk was pedestrians, and the particulate matter emitted by vehicle exhaust is greatly reduced compared with the main road. Therefore, the dust accumulation of *Euonymus japonicus* planted in different locations of urban streets was significantly different.

### Response of leaf functional traits to urban atmospheric particulate matter

It was found that the plant functional traits change regularly with the change of atmospheric particulate matter. In general, with the increase of atmospheric particulate matter, leaf thickness (LT), leaf dry matter content (LDMC), leaf tissue density (LTD) and stomatal density (SD) all increased, while the chlorophyll content index (CCI) and specific leaf area (SLA) decreased.

**Fig. 2:**
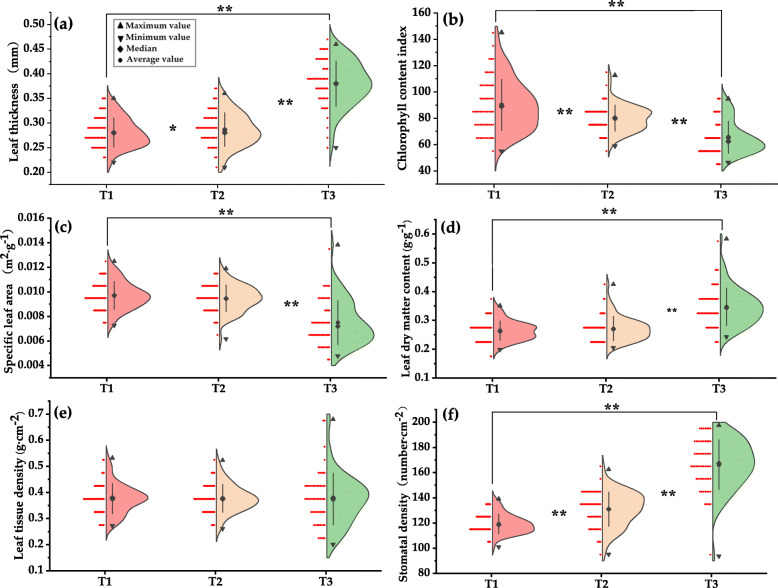
Plant functional traits under the influence of different amounts of dust deposition. Figures (a)-(f) are leaf thickness, chlorophyll content index, specific leaf area, leaf dry matter content, leaf tissue density, and stomatal density. T1: outer sidewalk area (slightly polluted area of atmospheric particulate matter), T2: area between the relief road and the sidewalk (moderately polluted area of atmospheric particulate matter), T3: area in the middle of the main road (heavy pollution area of atmospheric particulate matter)

As shown from Fig. 2a, compared with T1, the leaf thickness of T2 and T3 were significantly increased, and the difference reached a significant level (*p* < 0.05) and an extremely significant level (*p* < 0.01). Leaf thickness is generally considered as a valuable characteristic, which may be related to resource acquisition, water conservation and assimilation [41]. Leaf tissue thickness was positively correlated with leaf water use efficiency, and is also closely related to leaf water storage capacity [42]. Studies have shown that the increase in leaf thickness or density was beneficial to increase the distance or resistance of water diffusion from the inside of the leaf to the leaf surface, and reduce the internal water loss in the plants [43]. In this study, we found that with the increase of atmospheric particulate matter pollution, the leaf thickness showed a significant increasing trend. This indicates that plants can save water by increasing the leaf thickness in an environment polluted by atmospheric particles.

As shown from Fig. 2b, the chlorophyll content index in T2 and T3 decreased significantly, and reached an extremely significant level between T1, T2 and T3 (*p* < 0.01). Chlorophyll content index reflects the photosynthesis ability of plants to some extent [44]. In this study, the chlorophyll content index was significantly reduced by atmospheric particles. We suspect, on the one hand, it may be related to the dust falling on the leaf. A large amount of dust was attached to the blade surface, which covers the contact area of the blade and the outside, especially the leaf area used for photosynthesis. On the other hand, the decrease of chlorophyll content may also result from the allocation of plant resources. Under limited resources, plants use more resources to build defensive structures, thus weakening the resources for photosynthesis.

As shown from Fig. 2c, the specific leaf area of T3 was significantly more extensive than that of T1 and T2 (*p* < 0.01), but there was no significant difference between T1 and T2. Specific leaf area is closely related to the growth and survival strategies of plants, which can characterize the adaptability of plants to the environment and the ability to obtain resources [26, 45–47]. Studies have shown that plants with a lower leaf area can better adapt to resource-poor and arid environments, while plants with a higher leaf area have a better ability to retain nutrients in the body [26, 48]. In this study, we found that the specific leaf area of plants generally decreases with the increase of urban atmospheric particulate matter. Reducing the specific leaf area of plants was very beneficial to the light energy capture by leaves. At the same time, the harm caused by atmospheric particulate matter was reduced, and the adaptability to environmental pollution is enhanced. Therefore, the relatively low specific leaf area of urban plants in areas with high atmospheric particulate matter resulted from their long-term adaptation to urban atmospheric particulate matter pollution. It was also their long-term survival strategy in a polluted environment.

As shown from Fig. 2d, the leaf dry matter content of T3 was significantly higher than that of T1 and T2 (*p* < 0.01), but there was no a significant difference between T1 and T2. Leaf dry matter content can reflect the adaptability of leaves to arid climates, and is the most stable variable on the axis of resource acquisition [49]. Leaf dry matter content is the preferred index in plant ecology research, and it can be a good indicator of the plant’s ability to preserve nutrients [43, 50]. In this study, it was found that there were significant differences in dry matter content of leaves under the urban atmospheric particulate matter, which increased with the increase of pollution. With the increase of leaf dry matter content, the distance or resistance of water diffusion from leaves to leaf surfaces was increased, and the water loss in the plants was reduced. Therefore, under the influence of atmospheric particles, the dry matter content in the leaves of plants was higher, which may increase their environmental adaptability. At the same time, it also shows that under the influence of atmospheric particulate matter, *Euonymus japonicus* uses more nutrients to build its defense structure and supported structure.

As shown from Fig. 2e, leaf tissue density of T3 was higher than that of T1 and T2, but there was no significant difference. Studies have shown that the increase in leaf tissue density is beneficial to reducing transpiration, thus reducing water loss of plants [43]. Meanwhile, the increase in leaf tissue density can slow down the growth of plants and store more carbon for the construction of defense organizations [46, 48]. In this study, leaf tissue density showed an increasing trend with the aggravation of atmospheric particulate matter pollution. This shows that plants strengthen their defense structure to reduce the damage of atmospheric particles to leaves. This was similar to the response strategy of plants to high temperature and drought [43].

As shown from Fig. 2f, compared with T1, the stomatal density of T2 and T3 increased significantly (*p* < 0.01). Stomatal is an essential window for plants to exchange gas and moisture outside [50, 51]. This study shows that atmospheric particulate matter directly impacts on stomatal density, and the aggravation of pollution promotes the increase of stomatal density. As atmospheric particles were deposited on the blade surface, some air holes may be blocked, leading to an imbalance of the blade’s ability to exchange gas with the outside world. Therefore, increasing the stomatal density of plants may be a regulation strategy to deal with atmospheric particulate matter.

### Influence of atmospheric particulate matter on the spatial distribution of stomata

**Fig. 3:**
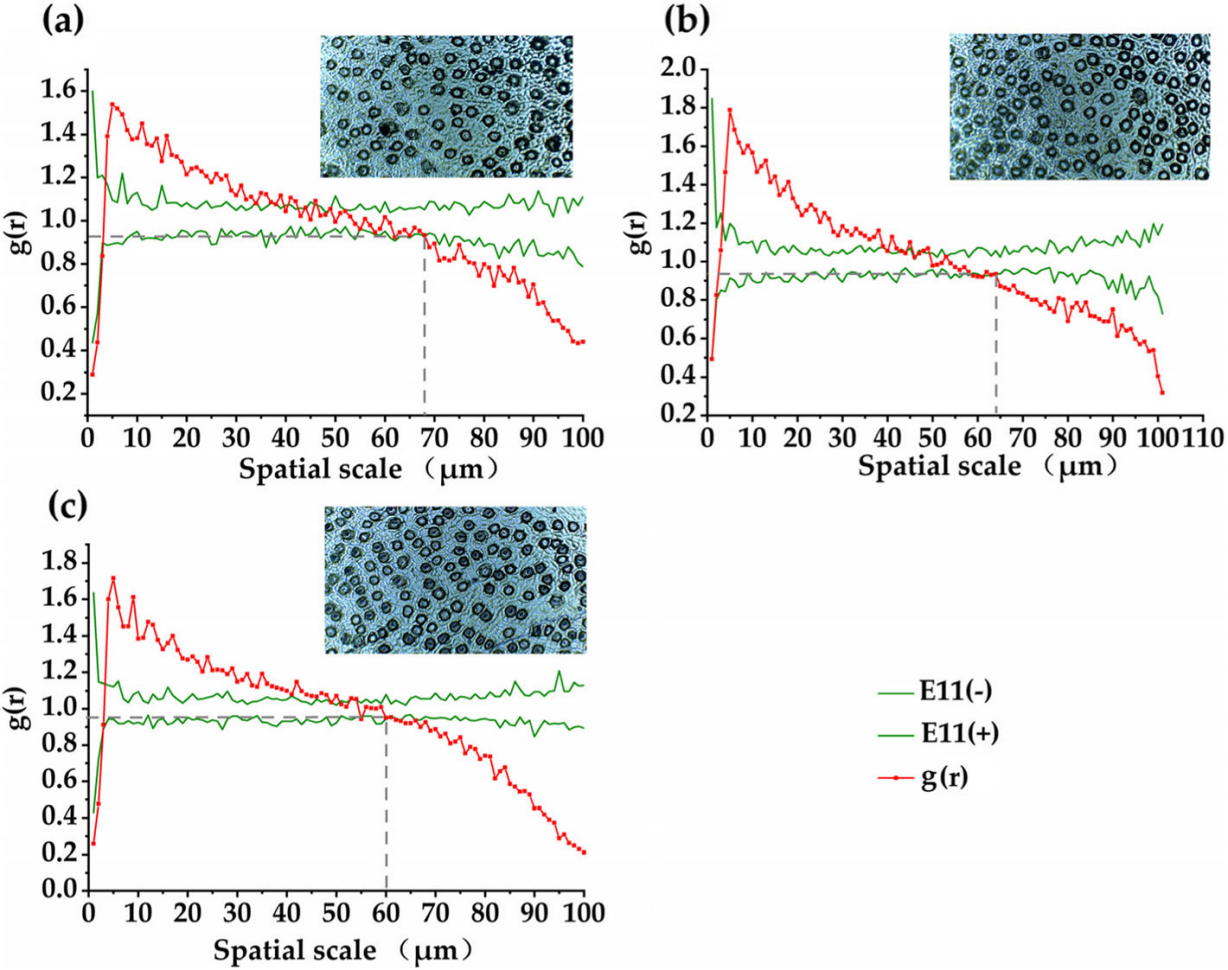
Effect of atmospheric particulate matter on spatial distribution pattern of stomata in *Euonymus japonicus* leaves. (a)-(c) respectively represent T1, T2 and T3. T1: outer sidewalk area (slightly polluted area of atmospheric particulate matter), T2: area between the relief road and the sidewalk (moderately polluted area of atmospheric particulate matter), T3: area in the middle of the main road (heavy pollution area of atmospheric particulate matter). E11(−): upper envelope trace, E11(−): lower envelope trace, g(r): paired correlation function

As shown in Fig. 3, the distribution characteristics of the stomatal spatial pattern of *Euonymus japonicus* were obviously different in different atmospheric particulate matter environments. The stomata of *Euonymus japonicus* were aggregated at the scales of 0 ~ 42.65 (±7.84) μm (T1), 0 ~ 46.09 (±9.54) μm (T2) and 0 ~ 54.93 (±16.21) μm (T3), and distributed randomly at the scales of 42.65 (±12.61) ~ 69.54 (±21.02) μm (T1), 46.32 (±17.69) ~ 64.74 (±25.53) μm (T2) and 54.83 (±17.80) ~ 60.64 (±28.31) μm (T3), and distributed randomly at the scales of 69.45 (±24.42) ~ 100 μm. This indicates that atmospheric particulates change the spatial distribution pattern of stomata of *Euonymus japonicus*, and the spatial scale of stomata changes from random distribution to uniform distribution under different atmospheric particulates. With the increase of interception of atmospheric particulate matter in leaves, the spatial distribution pattern of stomata of *Euonymus japonicus* becomes more regular. We suspect that this may be a regulatory strategy adopted by plants to treat the atmospheric particulate matter. *Euonymus japonicus* can prevent the influence of atmospheric particulate matter on leaf water diversion by adjusting the distribution pattern of stomata.

### Correlation between leaf functional traits and atmospheric particulate matter

**Fig. 4:**
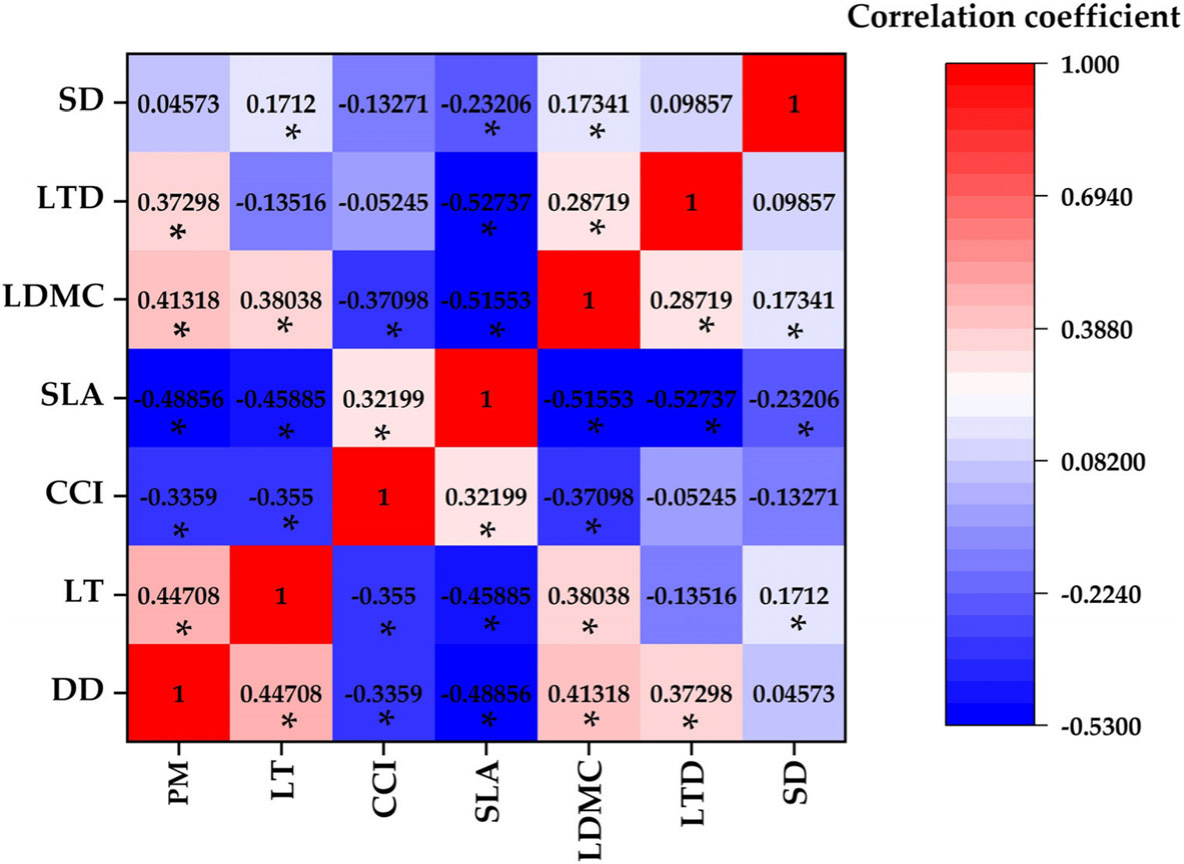
Correlation between leaf functional traits of *Euonymus japonicus* in the environment polluted by atmospheric particulate matter. PM: atmospheric particulate matter, LT: leaf thickness, CCI: chlorophyll content index, SLA: specific leaf area, LDMC: leaf dry matter content, LTD: leaf tissue density, SD: stomatal density. “*” indicate that the indicators have reached a significant difference at the *p* < 0.05 level

The leaf thickness, leaf dry matter content, leaf tissue density and stomatal density were positively correlated with atmospheric particulate matter. However, chlorophyll content index, specific leaf area and atmospheric particulate matter were negatively correlated (Fig. 4). The Pearson correlation coefficient values from large to small were specific leaf area (− 0.4885), leaf thickness (− 0.4471), leaf dry matter content (0.4132), leaf tissue density (0.3730), chlorophyll content index (0.3359) and stomatal density (0.0457). Except for stomatal density, other plant traits were significantly related to atmospheric particulate matter. Among them, the response of specific leaf area to atmospheric particulate matter was the most severe.

### Correlation and synergistic relationship between leaf functional traits of *Euonymus japonicus*

**Fig. 5:**
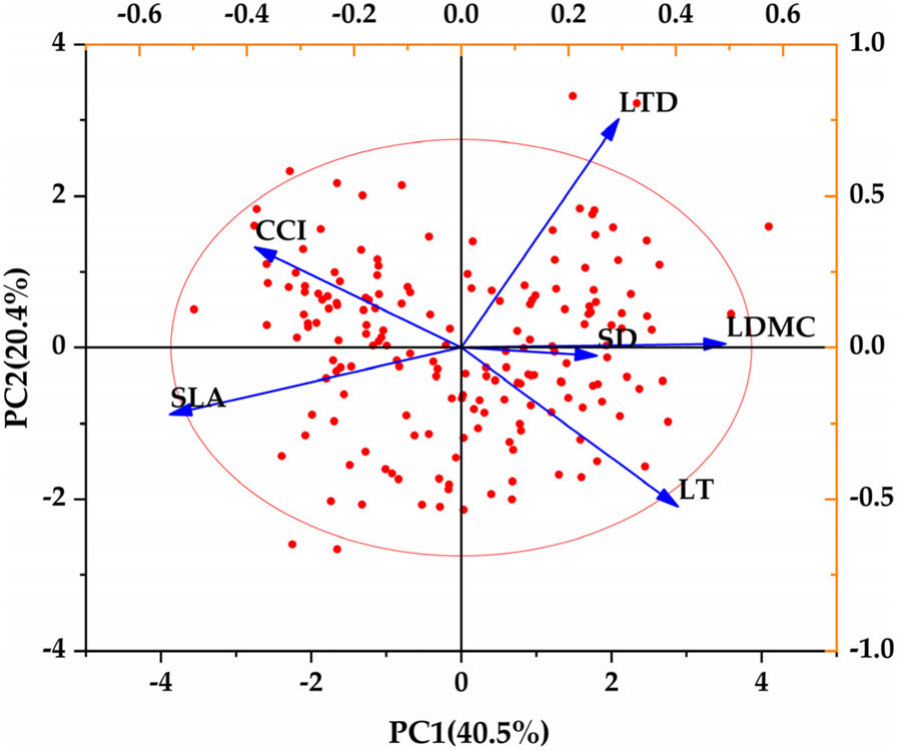
Principal component analysis biplot of leaf functional traits. LT: leaf thickness, CCI: chlorophyll content index, SLA: specific leaf area, LDMC: leaf dry matter content, LTD: leaf tissue density, SD: stomatal density

It can be seen from Fig. 5 that there was a specific quantitative relationship between different functional traits due to environmental pressure (atmospheric particulate matter) and plant trade-off strategy. Stomatal density was positively correlated with leaf thickness and dry matter content. The leaf tissue density was negatively correlated with specific leaf area, and positively correlated with leaf dry matter content. The leaf dry matter content was negatively correlated with leaf thickness, chlorophyll content index and specific leaf area. The specific leaf area was negatively correlated with leaf thickness, and positively correlated with chlorophyll content index. The leaf thickness was negatively correlated with the chlorophyll content index.

As shown in Table 1, two principal components were extracted according to the principle that the eigenvalue was greater than 1 (the eigenvalues were 2.429 and 1.226, respectively). The contribution rates of these two principal components were 40.5 and 20.4%, respectively, and the cumulative contribution rate was 60.9%, which indicates that these two principal components were the main factors in the change of leaf functional traits. The initial factor loading matrix of the principal components (Table 1) and the loading diagram of principal component analysis (PCA) were used, it can be seen that leaf thickness, leaf dry matter content, leaf tissue density and stomatal density were significantly positively correlated with the first principal component, and chlorophyll content index and specific leaf area were significantly negatively correlated with the first principal component. The correlation (absolute value) size was SLA > LDMC > LT > CCI > LTD > SD. Principal component analysis showed that the indicators significantly related to the first principal component could be used as the main indicators of leaf functional traits. The principal component contrasts leaf area, leaf dry matter content and leaf thickness have the greatest variation.

**Table 1:** Factor matrix and principal component contribution rate of leaf functional traits. LT: leaf thickness, CCI: chlorophyll content index, SLA: specific leaf area, LDMC: leaf dry matter content, LTD: leaf tissue density, SD: stomatal density

Projects	Scores	
	PC1	PC2
Eigenvalues	2.429	1.226
Cumulative contribution rate /%	40.483	20.436
LT	0.404	−0.524
CCI	−0.385	0.331
SLA	−0.544	−0.220
LDMC	0.493	0.012
LTD	0.293	0.753
SD	0.253	−0.028

### The trade-off strategy of plant functional traits on atmospheric particulate matter and analysis of leaf economics spectrum at the intraspecific level

Generally, the total resources available to plants are limited. If plants invest more resources in a certain functional trait, it will inevitably reduce its investment in other traits, that is, at the expense of the construction and functional maintenance of other traits [52–54]. In other words, in a limited resource environment, plants will optimize resource allocation among functional traits [53]. In this study, due to the pressure of atmospheric particulate environment, *Euonymus japonicus* showed a trade-off strategy at the leaf level, which constituted a complex and orderly trade-off relationship of economics spectrum characteristics. *Euonymus japonicus* will adjust, transform or compensate its functions according to its resource conditions in the urban environment, to achieve and balance the three purposes of “survival, growth and reproduction”, which is finally manifested in the functional characteristics of plant leaves. For example, due to the influence of atmospheric particulate matter, the specific leaf area is significantly reduced, which indicates that plants may use a large part of materials to build protective structures, so as to reduce the damage of atmospheric particles to leaves. The decrease of specific leaf area indicates that the greater the leaf area per unit mass of the plant, the thicker the leaf is. At this time, the more carbon the leaves were used to build the protective structure and supporting structures. It is manifested by increasing leaf dry matter content and leaf tissue density at the trait level.

**Fig. 6:**
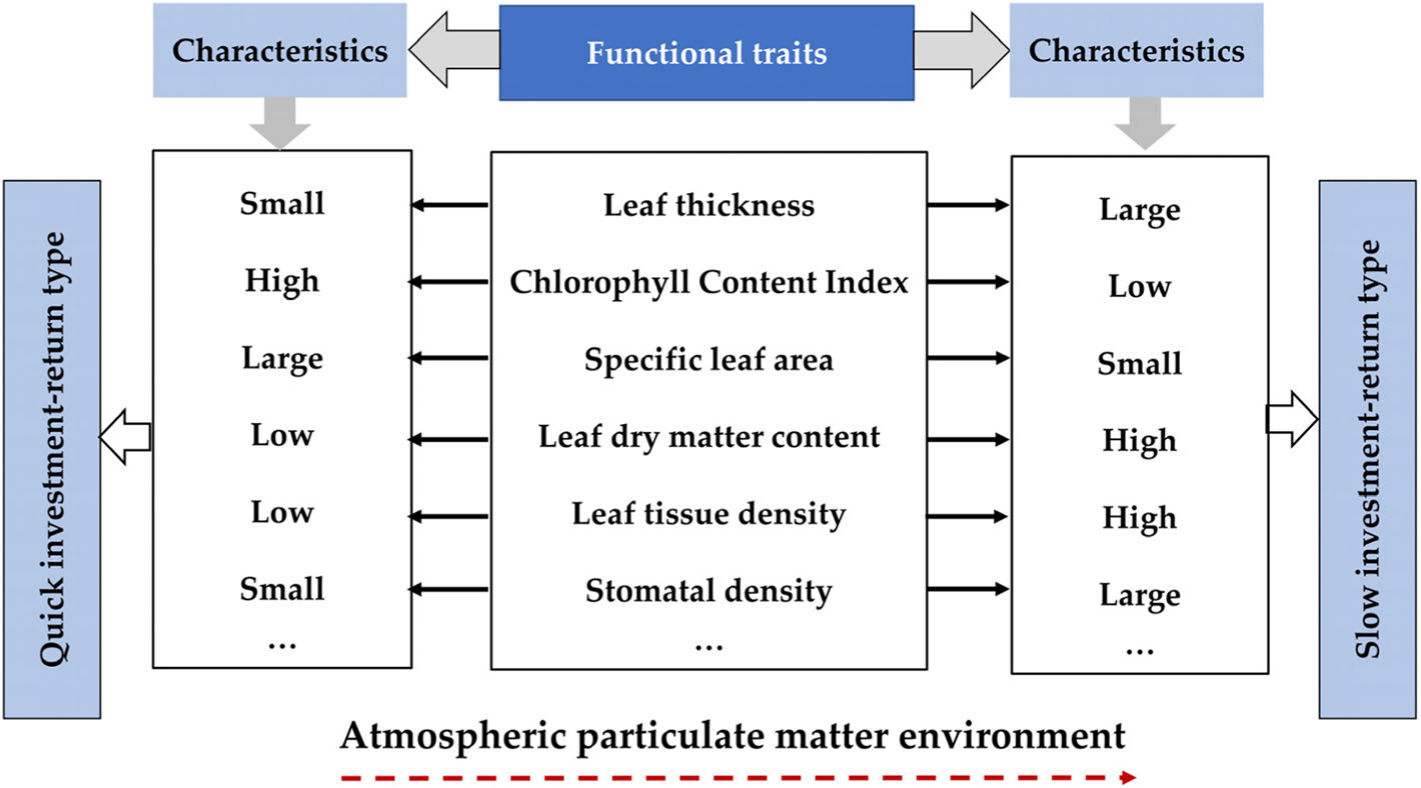
Leaf economics spectrum (intraspecific level) of *Euonymus japonicus* under atmospheric particulate matter environment

It was pointed out that the leaf economics spectrum was a series of interrelated and coordinated combinations of functional characteristics, and it also quantitatively represents a series of regular and changing strategies of plant resource balance [54–58]. At one end of this economic pedigree, it shows the ability of “quick investment-return”, while at the other end, it shows the ability of “slow investment-return” [48, 55]. On the whole, under the influence of urban atmospheric particulate matter, *Euonymus japonicus* shows a “slow investment-profit” type in the global leaf economics spectrum, which has low specific leaf area, low chlorophyll content index, large leaf thickness, high leaf dry matter content, leaf tissue density and stomatal density (Fig. 6).

## Conclusions

This study discussed the response of leaf functional traits of *Euonymus japonicus* to atmospheric particulate matter, and provided the theoretical basis for monitoring and predicting the impact of future environmental pollution changes on plants and ecosystems. The analysis leads to the following conclusions.

1. With the increase of atmospheric particulate matter, leaf thickness, leaf dry matter content, leaf tissue density and stomatal density increased, while chlorophyll content index and specific leaf area decreased. Under the influence of atmospheric particulate matter, *Euonymus japonicus* can improve its gas exchange efficiency by optimizing the spatial distribution characteristics of stomata. The larger the atmospheric particulate matter, the more regular the spatial distribution pattern of stomata.
2. The leaf thickness, leaf dry matter content, leaf tissue density and stomatal density were positively correlated with atmospheric particulate matter. However, chlorophyll content index and specific leaf area were negatively correlated with atmospheric particulate matter. The response of specific leaf area to atmospheric particulate matter was the most significant.
3. In the urban atmospheric particulate environment, *Euonymus japonicus* showed a trade-off strategy at the interspecific level, which constituted a complex and orderly trade-off relationship of economics spectrum characteristics. *Euonymus japonicus* shows a “slow investment-return” type in the leaf economics spectrum at the interspecific level, which is characterized by low specific leaf area, low chlorophyll content index, large leaf thickness, large stomatal density, high leaf dry matter content and high leaf tissue density.

At the leaf level, leaf functional traits may change among populations, the resource use strategy shifting to best suit the current environmental conditions. Leaf functional traits change in the environment of atmospheric particulate matter. The resource use strategy was shifting to best suit the current urban environment. Our study found that leaf functional traits in *Euonymus japonicus* covaried in patterns consistent with the leaf economics spectrum. This discovery provides a profound understanding of the adaptation of leaf functional traits under the background of urban environmental change.

## Materials and methods

### Sample plot setting and sampling

**Fig. 7:**
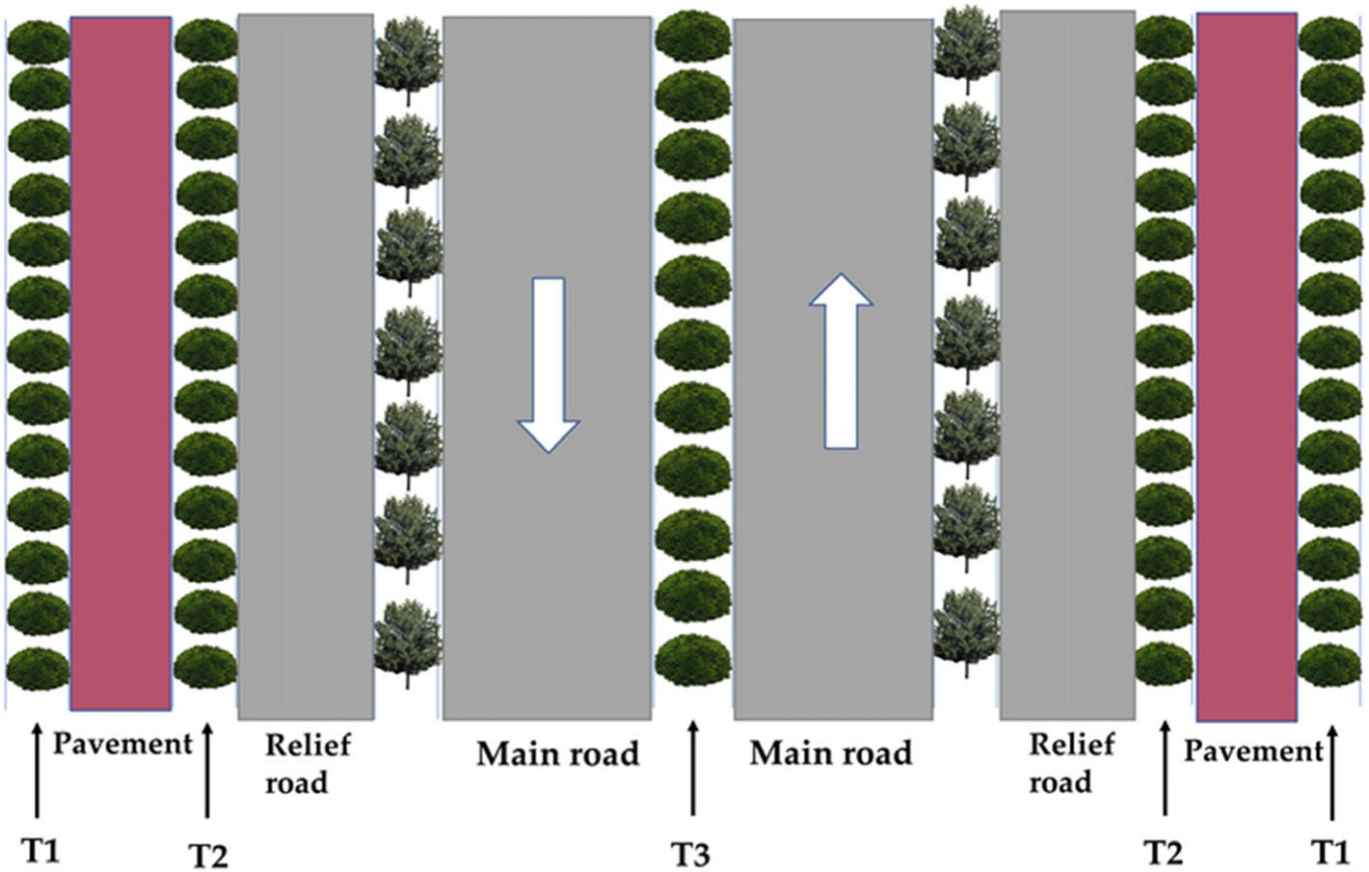
Sampling position diagram of leaf samples. T1: outer sidewalk area (slightly polluted area of atmospheric particulate matter), T2: area between the relief road and the sidewalk (moderately polluted area of atmospheric particulate matter), T3: area in the middle of the main road (heavy pollution area of atmospheric particulate matter)

As shown in Fig. 7, according to the characteristics of road dust, we divided the pollution degrees of atmospheric particles matter according to the distance from the main road. In September, 2019, the leaves of *Euonymus japonicus* were collected from the outside of the sidewalk (T1), between the sidewalk and relief road (T2), and the center of the main road (T3). There was no rainstorm within 15 days on the sampling day to avoid the influence of rain erosion on dust deposition. The plants in this study are 6-year-old and have been planted in this area for 5 years. Average tree height is 1.75(±0.56) m. We selected Sixty plants for each treatment, and randomly collected three mature and healthy leaves from the middle canopy of each tree. A total of 180 leaves were collected for each treatment. And then, the leaves were placed in a tray and sealed with plastic wrap. The leaf surface was not touched during the whole collection process.

### Dust deposition measurement

**Fig. 8:**
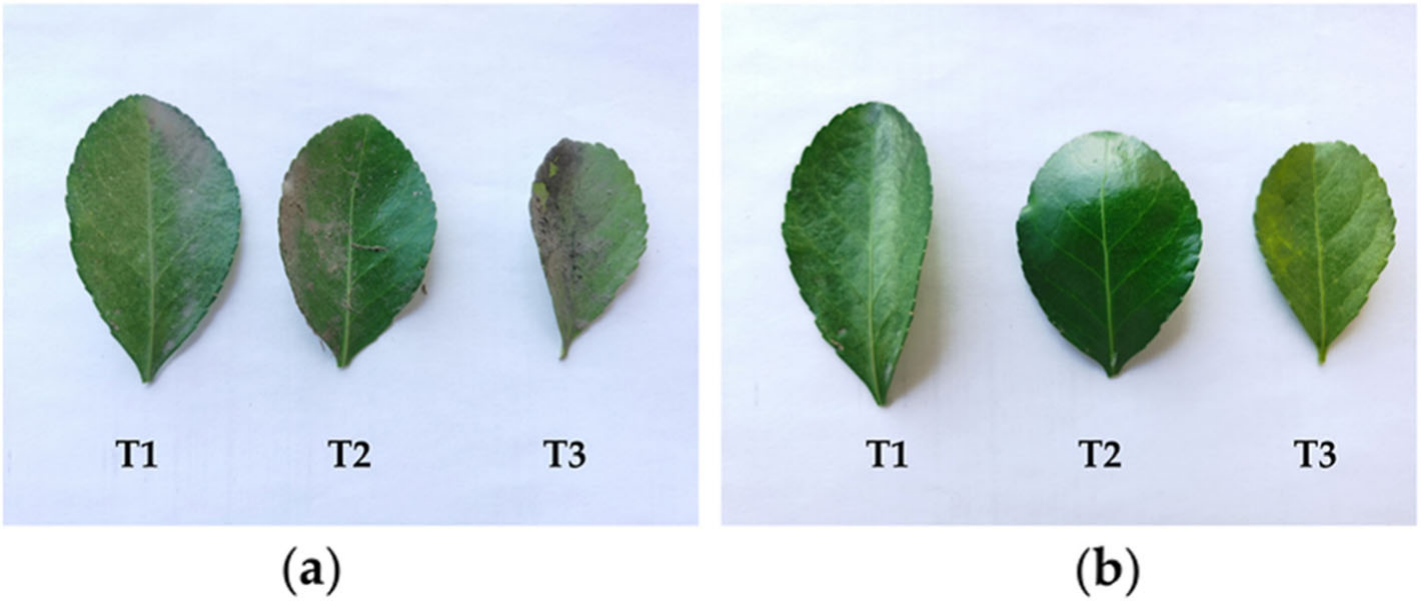
Samples of *Euonymus japonicus* leaves before (a) and after (b). (a) leaves before cleaning, and (b) leaves after cleaning. T1: outer sidewalk area (slightly polluted area of atmospheric particulate matter), T2: area between the relief road and the sidewalk (moderately polluted area of atmospheric particulate matter), T3: area in the middle of the main road (heavy pollution area of atmospheric particulate matter). Plant identification by Prof. Chengyang Xu from Beijing Forestry University (identification information refers to Flora of China)

After numbering the slow quantitative filter paper, weigh it (W_1_) with a GH-252 electronic balance (accuracy 0.1 mg, Shanghai Youyi Instrument Co., Ltd., Shanghai, China) for later use. Filter the washed liquid with slow quantitative filter paper, and then the dry the filter paper to constant weight by DHG-9143 electric heating blast drying cabinet (Shanghai Yitian Scientific Instrument Co., Ltd., Shanghai, China) at a temperature of 60 °C (the difference between the two measured values does not exceed 0. 0002 g). After that, dry filter paper (W_2_) was weighed. At the same time, we calculated the quality difference(△*W*) of the blank control filter paper before and after drying (△*W* = *W*_CK2_-*W*_CK1_, *W*_*CK1*_ is the value of the blank control filter paper before drying, *W*_*CK2*_ is the value of the blank control filter paper after drying). *W (*Total dust deposition*)* = *W*_*2*_-*W*_*1*_-*△W*. And then, sharp-nosed medical tweezers were used in the sampling and measuring process to avoid direct contact with the blade surface. The blades before and after cleaning were shown in Fig. 8.

### Leaf functional traits measurement

The wight of fresh leaves (LFW) were weighed with JA1003N one-thousandth electronic balance (±0.001 g, Shanghai Jinghai Instrument Co., Ltd., Shanghai, China), and the measurement was completed within 30 min after removal. The leaf area (LA) was measured and automatically calculated with the V39 portable leaf area meter (±0.01 mm^2^, Seiko Epson Corporation, Shanghai, China). Leaf thickness (LT) was measured with 500–196-30 vernier calipers (±0.01 mm, Suzhou Quantum Instrument Co., Ltd., Suzhou, China), and the thickness was measured at three equidistant points along the main vein of the leaf about 0.25 cm. The thickness of each position was averaged as the leaf thickness of the blade. The leaf volume (V_L_) was measured by the drainage method. First, put an appropriate amount of distilled water (V_1_) in a 500 mL graduated cylinder, and then immerse the leaf in water (V_2_). The volume difference between the front and back was the volume of the leaf V_L_ = V_2_-V_1_. Soak the leaves in deionized water and place them in a dark refrigerator at 5 °C for 12 h. Take out the leaves and use absorbent paper to absorb the water on the surface of the leaves and wipe the impurities on the leaves, and then weigh the leaves saturated fresh weight (LSFW). Then dry at 60 °C to constant weight (48 h), and weigh the dry mass (LDM).

1 $$ \mathrm{LTD}=\mathrm{LDM}/{\mathrm{V}}_{\mathrm{L}} $$

2 $$ \mathrm{SLA}=\mathrm{LA}/\mathrm{LDM} $$

3 $$ \mathrm{LDMC}=\mathrm{LDM}/\mathrm{LSFW} $$

Temporary slide of stomata was made by imprinting method. A layer of transparent imprinting liquid was evenly coated on the backside of the leaf from the base to the tip of the leaf, avoiding the main vein, and the imprinting film was turn off with tweezers to make temporary slides. Three slides were made for each leaf, and each slide was magnified by XSP-20 optical microscope (Jiangnan Yongxin Optics Co., Ltd., Nanjing, China) and then randomly selected 5 fields of view (713.191 μm × 958.115 μm) for image acquisition. The stomatal density was calculated using image J software.

### Point pattern analysis of stomata

This analysis considers that each stomatal was a single point distributed on the blade surface, and the middle position of the stomatal opening was the position of this single point. Firstly, the selected micrographs were digitized under the same coordinate system by using the spatial distribution software ArcGIS 10.4, and the coordinate values of each pore of the selected photographs can be obtained. Then, the stomatal pattern was analyzed by using Programita Febrero 2014. Here, we used Ripley’s K-Function, a spatial statistical analysis method, to carry out spatial analysis on the digitized stomatal distribution feature point. Ripley’s K-Function is a kind of distribution accumulation function, which uses the second-order matrix of all single points distances to explore the two-dimensional distribution patterns of these points on different scales. This paper adopted the paired correlation function g(r) and Ripley’s K(r) function. The g(r) function is derived from the K(r) function. The K(r) function is defined as taking any point as centering of the circle. “r” is the ratio of the expected number of points in the circle with the radius to the point density in the sample square. In the g(r) function, a circle is used to replace the circle in the traditional pattern analysis. Among them, the g(r) function can explain the neighbor density and eliminate the cumulative effect of the K(r) function, so the g(r) function is more intuitive than the cumulative calculation of the K(r) function. The expression of g(r) function is as follows [59].

4 $$ \mathrm{g}\left(\mathrm{r}\right)=\frac{\mathrm{K}\left(\mathrm{r}\right)}{2\uppi \mathrm{r}}\ \left(\mathrm{r}\ge 0\right) $$

Under the assumption of complete spatial randomness, the g(r) value is above the envelope trace, indicating that the stomata are clustered and distributed on the r scale. The g(r) value is located between the envelope traces, indicating that the stomata are randomly distributed on the r scale. The g(r) value is below the envelope trace, indicating that the stomata are uniformly distributed on the r scale.

### Data analysis

All the data were sorted in Excel 2020, and the data were analyzed and drawn by Origin 2019b. One-way ANOVA and least square multiple comparison were used to test the significance of differences in leaf functional traits under different atmospheric particle gradients. Pearson correlation analysis was used to analyze the internal correlation of plant functional traits and the correlation between plant functional traits and atmospheric particulate matter.

## Data Availability

The data involved in the article were all shown in the figures and tables. However, there are still available from the first author on reasonable request.
